# Intraspecific variation in defense against a generalist lepidopteran herbivore in populations of *Eruca sativa* (Mill.)

**DOI:** 10.1002/ece3.1805

**Published:** 2016-01-01

**Authors:** Ariel Ogran, Netanel Landau, Nir Hanin, Maggie Levy, Yedidya Gafni, Oz Barazani

**Affiliations:** ^1^ Institute of Plant SciencesAgricultural Research OrganizationThe Volcani CenterBet Dagan50250Israel; ^2^ The Mina & Everard Goodman Faculty of Life SciencesBar Ilan UniversityRamat Gan5290002Israel; ^3^ The Robert H. Smith Faculty of AgricultureFood and EnvironmentThe Hebrew University of JerusalemRehovot76100Israel

**Keywords:** Glucosinolate, induced defense, isothiocyanate, jasmonic acid, nitrile, proteinase inhibitor

## Abstract

Populations of *Eruca sativa* (Brassicaceae) from desert and Mediterranean (Med) habitats in Israel differ in their defense against larvae of the generalist *Spodoptera littoralis* but not the specialist *Pieris brassicae*. Larvae of the generalist insect feeding on plants of the Med population gained significantly less weight than those feeding on the desert plants, and exogenous application of methyl jasmonate (MJ) on leaves of the Med plants significantly reduced the level of damage created by the generalist larvae. However, MJ treatment significantly induced resistance in plants of the desert population, whereas the generalist larvae caused similar damage to MJ‐induced and noninduced plants. Analyses of glucosinolates and expression of genes in their synthesis pathway indicated that defense in plants of the Med population against the generalist insect is governed by the accumulation of glucosinolates. In plants of the desert population, trypsin proteinase inhibitor activity was highly induced in response to herbivory by *S. littoralis*. Analysis of genes in the defense‐regulating signaling pathways suggested that in response to herbivory, differences between populations in the induced levels of jasmonic acid, ethylene, and salicylic acid mediate the differential defenses against the insect. In addition, expression analysis of myrosinase‐associated protein *NSP2* suggested that in plants of the desert population, glucosinolates breakdown products were primarily directed to nitrile production. We suggest that proteinase inhibitors provide an effective defense in the desert plants, in which glucosinolate production is directed to the less toxic nitriles. The ecological role of nitrile production in preventing infestation by specialists is discussed.

## Introduction

In the Brassicaceae and its closely related plant families, the sulfur‐ and nitrogen‐containing glucosinolates (GS) form the predominant defense against herbivores and pathogens. Derived from amino acids, GS are categorized as aromatic, indolic, or aliphatic, resulting in more than 100 compounds that differ from each other in the structure of a side chain (Agerbirk and Olsen 2012). Damage by herbivores releases the enzyme myrosinase which hydrolyzes the glucose residue, forming the bioactive metabolites isothiocyanates (ITC), thiocyanate, epithionitriles, or nitriles. While ITCs are the default breakdown products of myrosinase activity, that of thiocyanate, epithionitriles, and nitriles depend on specifier proteins, that is, thiocyanate‐forming protein, epithiospecifier protein (ESP), and nitrile‐specifier protein (NSP), respectively (Wittstock and Burow 2007). In *Arabidopsis*, an additional myrosinase‐associated protein, the epithiospecifier modifier (ESM), suppresses ESP expression, leading to increased hydrolysis of GS to ITC (Zhang et al. 2006).

Specialist and generalist herbivores both trigger GS synthesis and accumulation (Reymond et al. 2004; Bidart‐Bouzat and Kliebenstein 2011). However, specialist insects (e.g., *Pieris* sp., *Pluttela xylostella*) have evolved physiological means to overcome the toxic effects of GS; the deterrent and toxic properties of these metabolites are therefore mostly effective against generalists (Ratzka et al. 2002; Textor and Gershenzon 2009; Winde and Wittstock 2011). Numerous reports have shown natural intraspecific variation in GS concentrations (Windsor et al. 2005; Clauss et al. 2006; Bidart‐Bouzat and Kliebenstein 2008; Poelman et al. 2008); several have indicated negative correlations between the constitutive and induced concentrations of GS for defense against lepidopteran generalist insects (Clauss et al. 2006; Gols et al. 2008). Bidart‐Bouzat and Kliebenstein (2008) similarly associated intraspecific GS concentrations in 20 natural accessions of *Arabidopsis* with resistance against insects, but found a positive correlation between total GS concentration and the extent of the damage caused by specialists in field experiments.

In *Arabidopsis*, intraspecific variation in GS breakdown products has indicated a role for ITC and nitriles in the defense against herbivores. Allelic variation between the Columbia (Col) and Landsberg *erecta* (Ler) ecotypes resulted in functional activity of ESP in Ler, subsequently decreasing this ecotype's resistance to generalist herbivores relative to Col (Lambrix et al. 2001). This study by Lambrix et al. (2001) was followed by other reports on transgenic *Arabidopsis* lines, showing that generalist insects perform better on nitrile‐producing lines than on those that express ITC (e.g., Zhang et al. 2006; Burow et al. 2006b). However, natural variations in GS breakdown products that result in differences in defense against herbivores have so far been reported only in *Arabidopsis*.

**Figure 1:**
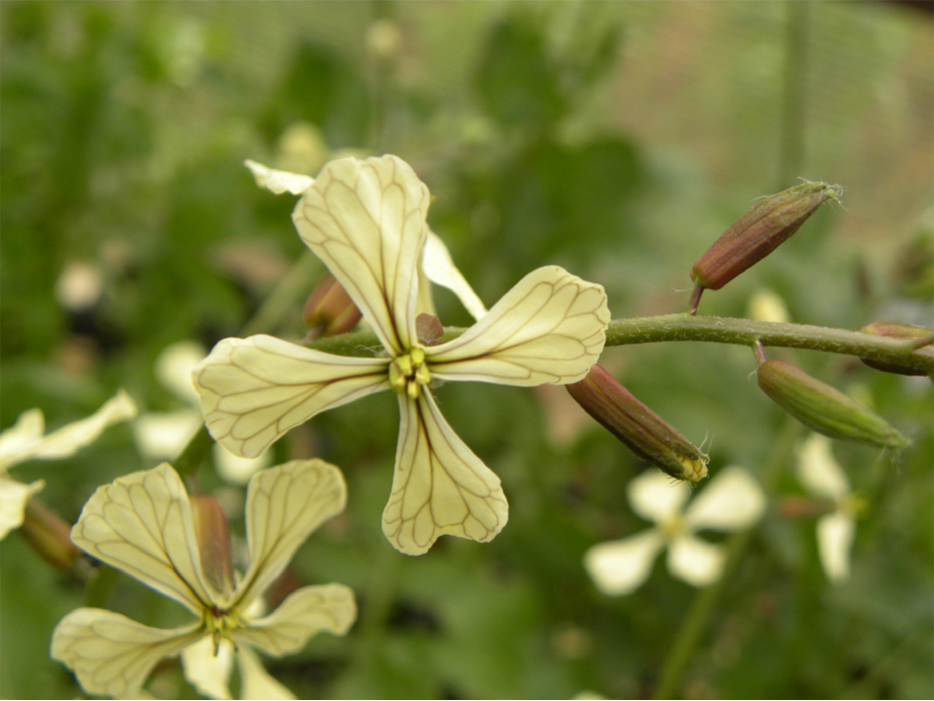
*Eruca sativa* Mill. (Brassicaceae) inflorescence, showing erect sepals and veined petals.

*Eruca sativa* Mill. (salad rocket, Brassicaceae) is a winter annual (Fig. 1), growing in nature mostly around the Mediterranean basin. Information that we have gathered in the last few years on wild populations of *E. sativa* indicates genetic differences between populations originating from the steep climatic gradient in Israel (Barazani et al. 2012; Westberg et al. 2013). Phenotypic evaluation in common‐garden experiments has shown that populations from the southern arid environments differ from those in the northern, more favorable mesic Mediterranean habitats in several phenological and morphological traits, including trichome density and herbivory damage (Westberg et al. 2013). Interestingly, the strong statistical association found between AFLP (amplified fragment length polymorphism) outlier loci and trichome density measured in an insect‐free environment was replaced with an association of these loci to the level of herbivore damage in a common‐garden field experiment (Westberg et al. 2013). Accordingly, we hypothesized that populations of *E. sativa* from arid (desert) and Mediterranean (Med) habitats differ in their induced defense response to herbivory. Here, using an eco‐genomic approach, we report on intraspecific variation in defense against generalist herbivore in two populations of *E. sativa*, representing the desert and Med environments.

## Materials and Methods

### Plant growth conditions

Seed lots representing the southern desert (32°04′49″ N, 35°29′46″ E, ≤200 mm annual rainfall) and northern Med (32°46′39″ N, 35°39′29″ E, 430 mm annual rainfall) populations, created under uniform conditions (Barazani et al. 2012), were used for the experiments. Seeds of the two populations were germinated on moistened Whatman No. 1 filter paper in 9‐cm Petri dishes and germinated in a growth chamber at 25°C with a 8/16‐h day/night photoperiod. Four‐day‐old seedlings were transferred to germination trays, and 2 weeks later, they were transplanted into 1‐L pots. Plants were grown in a potting soil mixture (Shacham, Givat Ada, Israel) containing 50% peat, 30% tuff, and 20% perlite (w/w) in a clean insect‐free net‐house and irrigated with an automatic irrigation system (150 mL/day). Plants at the vegetative stage, before bolting, were used in all experiments; the experiments were conducted between February and March, with average max/min temperatures of 20/14°C.

### Insects

Larvae of *Spodoptera littoralis* and *Pieris* sp. are commonly used as a model for generalist and specialist chewing insects, respectively (e.g., Agrawal and Kurashige 2003; Heidel and Baldwin 2004; Reymond et al. 2004; Clauss et al. 2006; Burow et al. 2006b; Rohr et al. 2012; Schweizer et al. 2013; Rasmann et al. 2015). Thus, we here used larvae of *S. littoralis* and *P. brassicae* to test plant defense response and resistance against lepidopteran insects in populations of *E. sativa*, regardless to their abundance in the natural habitats.

### No‐choice herbivore experiments

Five hatched larvae were placed in a polyethylene box (13.5 × 10.5 × 5.0 cm) and fed on fresh leaves collected from representative plants of each population. In *Arabidopsis*, the accumulation of GS and the arrangement of their breakdown products are differently regulated in different vegetative tissues (Brown et al. 2003; Wentzell and Kliebenstein 2008); thus, larvae were fed on one fresh leaf at the same phenological stage. To test for induced resistance, leaves were collected 2 days after induction of the defense mechanism with methyl jasmonate (MJ) (150 *μ*g in lanolin) and replaced with fresh leaf every second day; noninduced plants were used as controls. Larva weight was determined 10 days later to evaluate plant resistance. The experiment consisted of 15 plants of each population for each induction treatment, that is, control and MJ‐induced plants.

**Figure 2:**
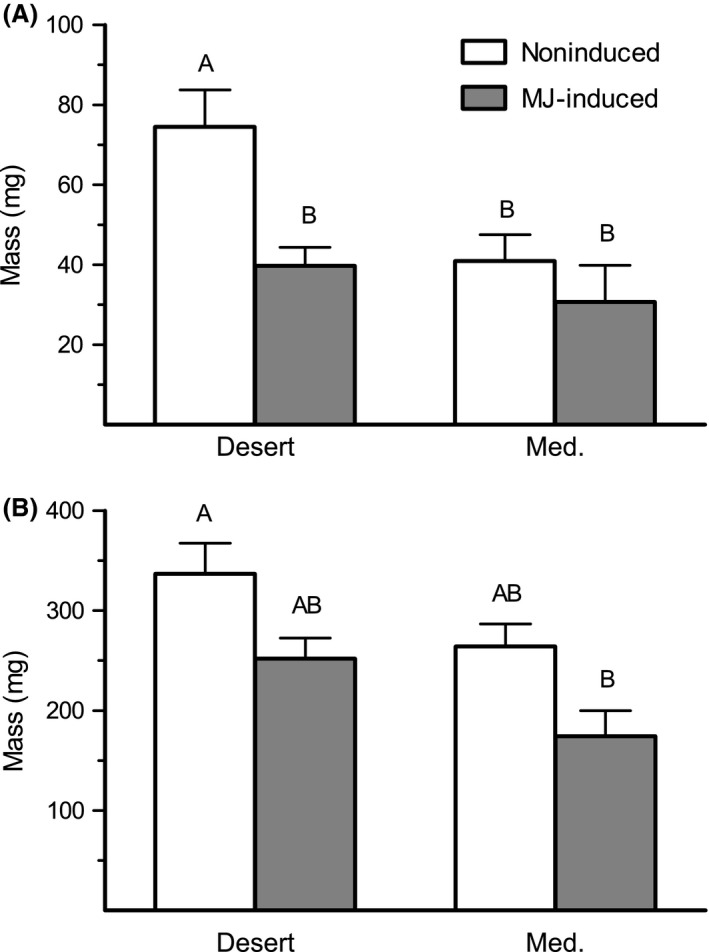
Mass gain of *S. littoralis* (A) and *P. brassicae* (B) feeding on noninduced and MJ‐induced *E. sativa* plants of the two investigated populations. Different letters above bars indicate significant differences (Tukey's HSD,* P *< 0.05).

Based on the results (Fig. 2), an additional experiment was conducted to evaluate the level of damage caused by *S. littoralis* larvae. Here, plants in their vegetative stage were randomly set up in the net‐house in two blocks: (1) control noninduced plants and (2) MJ‐induced plants. For induction, MJ (150 *μ*g in lanolin) was applied to two leaves, at 48‐h interval between induction treatments. Each of the two induction groups included 120 plants of each population. Three larvae of *S. littoralis*, at the first‐instar stage, were reared on each plant, and 7 days later, herbivore damage was recorded for each plant on a scale of 0 (no damage) to 5 (severe damage).

### Induction treatments

Defense response in plants at their vegetative stage, before bolting, was tested in three separate experiments: (1) following damage by herbivores, (2) after mechanical wounding, and (3) after MJ application. In the first, two larvae of either *S. littoralis* or *P. brassicae* were caged on one leaf at the same developmental stage in a polyethylene cup. In the two additional experiments, one leaf was induced by mechanical wounding with a pattern wheel or by application of MJ (150 *μ*g in lanolin). Samples were harvested at different time points from 0 (noninduced, control) to 48 h, time course that was shown to induce defense response and accumulation of GS in the Brassicaceae (e.g., Kliebenstein et al. 2002; Cipollini et al. 2003; Bidart‐Bouzat and Kliebenstein 2011; Badenes‐Perez et al. 2013). For each time point, one leaf was harvested from five to eight different plants. Samples were immediately frozen in liquid nitrogen and used for RNA isolation; samples harvested 48 h after damage by herbivores, MJ treatment, or wounding were also used for the analysis of defense metabolites.

### Glucosinolates extraction and HPLC analysis

Extraction and analysis of GS were performed as described previously by Mucha‐Pelzer et al. (2010) (Mucha‐Pelzer et al. 2010). Briefly, freeze‐dried 20‐ to 30‐mg samples were extracted with 70% methanol at 80°C, and the resulting GS‐containing extracts were applied on a Sephadex DEAE A25 anion‐exchange column (Sigma‐Aldrich, Israel); after several equilibration steps and overnight incubation with sulfatase, desulfo‐GS were eluted with 1‐mL aliquots of water, and the GS were separated and measured with an HPLC ProStar 240 high‐performance liquid chromatography (Varian, Palo Alto, CA) equipped with an Acclaim reverse‐phase C18 column (2.1 × 250 mm, 5 *μ*m) (Dionex, Sunnyvale, CA). The GS were quantified using benzyl GS as an internal standard.

### Proteinase inhibitor (PI) activity assay

Extraction of proteins from 100‐ to 150‐mg samples and analysis of PI activity followed a previously described method (Tremacoldi and Pascholati 2002; Sarmento et al. 2011) with BApNA (Nα‐benzoyl‐DL‐arginine‐4‐nitroanilide hydrochloride, Sigma‐Aldrich, Israel) as the substrate (Mueller and Weder 1989). Absorption of the extracts was measured at 405 nm; extraction buffer with or without standard soybean trypsin (*Glycine max*, Sigma‐Aldrich; 0.1 mg/mL in 1 mmol/L HCl) was used as a control. Based on Thaler et al. (1996), PI activity was calculated as the percentage of trypsin activity relative to controls (Thaler et al. 1996).

### RNA isolation and transcript analysis

RNA was isolated using the GeneJET purification kit (Thermo Scientific, MA, USA). Reverse transcription with oligo dT (Thermo Scientific) was used to synthesize cDNA. Based on orthologous sequences of *Arabidopsis* and *Brassica* available in GenBank databases, genes of interest from *E. sativa* were cloned and sequenced. Several key genes of interest, associated with plant defense response, were selected for the analysis: (1) *CYTOCHROME P450 79F1* and *79B3* (*CYP79F1* and *CYP79B3*), involved in the synthesis of aliphatic and indolic GS, respectively; (2) additional downstream, less specific GS postaldoxime enzyme S‐glycosyltransferase *UGT74B1* (Grubb and Abel 2006); (3) the myrosinase‐associated protein *NSP2*; (4) *ALLENE OXIDE CYCLYSE 1* (*AOC1*) and *ACC OXIDASE 1* (*ACO1*), key enzymes in the biosynthesis pathways of jasmonic acid (JA) and ethylene, respectively; and (5) *NONEXPRESSOR OF PATHOGENESIS‐RELATED PROTEINS 1* (*NPR1*), as a marker gene representing the synergistic crosstalk between JA‐ and salicylic acid (SA)‐signaling pathways (Pre et al. 2008).

Primer sets were designed on the basis of the obtained sequences, and RT‐PCR amplifications, using components supplied in the KAPA SYBR FAST kit (Kapa Biosystems, MA, USA), were performed on diluted cDNA samples of the same concentration (62 ng/*μ*L), to exclude primers that amplify paralogous genes. Gene‐specific primers (Table S1, Supporting Information) were then used in qRT‐PCR analysis on a Rotor‐Gene 6000 instrument (Corbett‐Qiagen, Valencia, CA) following a previously described protocol (Mayzlish‐Gati et al. 2010). The threshold cycle (Ct) was automatically determined by Rotor‐Gene 6000 software, and the relative expression levels of target genes were calculated using “two standard curves” (i.e., that of the gene of interest and that of actin) implemented in the Rotor‐Gene software. Each sample was analyzed in two technical replicates for each target gene. Standard curves were created in each run using a pooled cDNA sample; a reference cDNA calibrator sample was used to normalize the multirun results. Genes of the signaling‐regulating pathways were analyzed in samples that were collected after mechanical wounding and after damage by larvae of the generalist and specialist insects; the GS synthesis and myrosinase‐associated proteins were also analyzed after MJ treatment (above).

Preliminary experiment showed that application of pure lanolin did not have an effect on gene expression (data not shown).

### Data analysis

Raw data were subjected to statistical tests using the JMP v. 9.0.0 package (SAS Institute, Cary, NC). Analysis of variance (ANOVA) included analyses with repeated measures, two‐way ANOVA, and post hoc comparison using Tukey's HSD; post hoc *t*‐test was used for difference between means of two independent samples. All results are presented as the average mean of the biological replicates ± standard errors (SE).

## Results

### Defense against generalist and specialist herbivores in populations of *E. sativa*

Overall, the two populations differed in their resistance to larvae of *S. littoralis* and *P. brassicae*, and defense elicitation with MJ had an effect on resistance against the two herbivores (Table S2). The average weight of larvae of *S. littoralis* feeding on noninduced leaves of the desert population was significantly 1.8 higher than this feeding on noninduced leaves of the Med population (Fig. 2A). However, previous induction with MJ significantly increased the resistance of plants of the desert population to larvae of the generalist herbivore, but not in the Med ones (Fig. 2A; no significant effect of population × treatment, Table S2). In contrast to their effect on the generalist insect, no significant difference in the weight of the specialist *P. brassicae* was found between larvae feeding on MJ‐induced and noninduced plants of both populations (Tukey's HSD, Fig. 2B).

**Figure 3:**
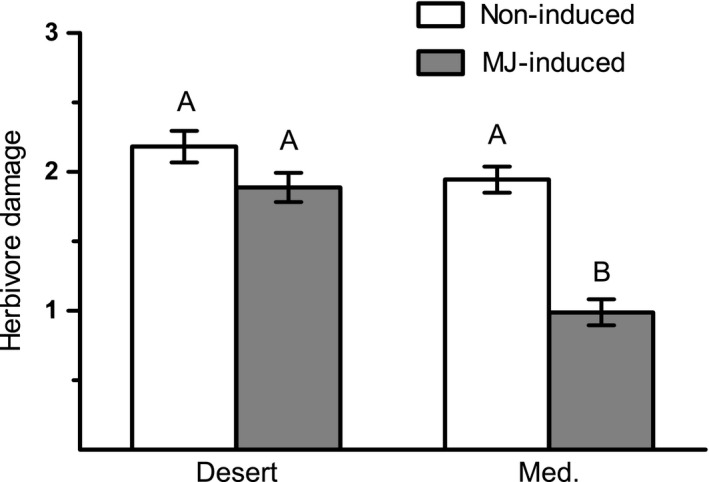
Estimated extent of the damage caused by larvae of *S. littoralis*. Larvae were reared on noninduced and MJ‐induced plants of the two populations, and the area of consumed leaves was qualitatively estimated on a scale ranging from no herbivory (0) to severe damage (5). Different letters above bars indicate significant differences (Tukey's HSD,* P <* 0.05).

Significant differences were found between plants of the two populations in the damage created by larvae of *S. littoralis* (Table S2). The larvae caused similar damage to noninduced plants of the two populations (Fig. 3). However, the average damage level in plants of the Med population was significantly reduced in MJ‐induced plants as compared to control plants (Tukey's HSD, *P *<* *0.05), which was not the case in plants of the desert population.

### Glucosinolates and gene expression analyses

**Figure 4:**
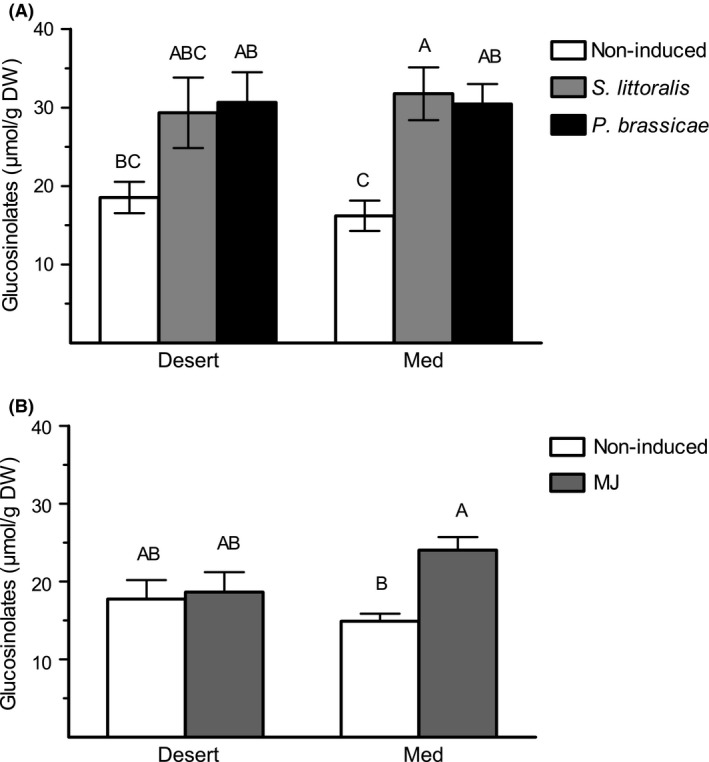
The concentration of total glucosinolates (*μ*mol/g DW, mean ± SE) in leaves of the two populations of *E. sativa*. Glucosinolates were analyzed in noninduced plants and 48 h after continuous elicitation by the generalist (*S. littoralis*) or specialist (*P. brassicae*) herbivores (A) or 48 h after MJ treatment (B). Different letters above bars indicate significant differences (Tukey's HSD,* P < *0.05).

No significant differences were found in the concentrations of total GS between noninduced (control) plants of the two populations. Defense elicitation with MJ or herbivory, either by larvae of the generalist or by specialist insects, shows an overall significant effect on the accumulation of GS (Table S3). Post hoc comparisons indicated that MJ and herbivory significantly induced the concentrations of total GS in plants of the Med population, but not in those of the desert population (Tukey's HSD, *P *<* *0.05; Fig. 4). Mechanical wounding and pure lanolin did not have any effect on GS accumulation in plants of the two populations (Fig. S1).

The concentrations of the desulfo‐GS are provided in the Supporting Information, Tables S4 and S5. Six main desulfo‐GS were detected in extracts of *E. sativa*; they were, in order of decreasing concentration: the aliphatic 4‐mercaptobutyl GS (glucosativin), 4‐methylthio‐3‐butenyl GS (glucoraphasatin), an as yet unidentified GS (X1), 4‐methylsulfinylbutyl GS (glucoraphanin), 4‐methylthiobutyl GS (glucoerucin), and the indolic 3‐indolylmethyl GS (glucobrassicin). A dimeric form of glucosativin (Bennett et al. 2002) was also identified (Tables S4 and S5, Supporting Information).

**Figure 5:**
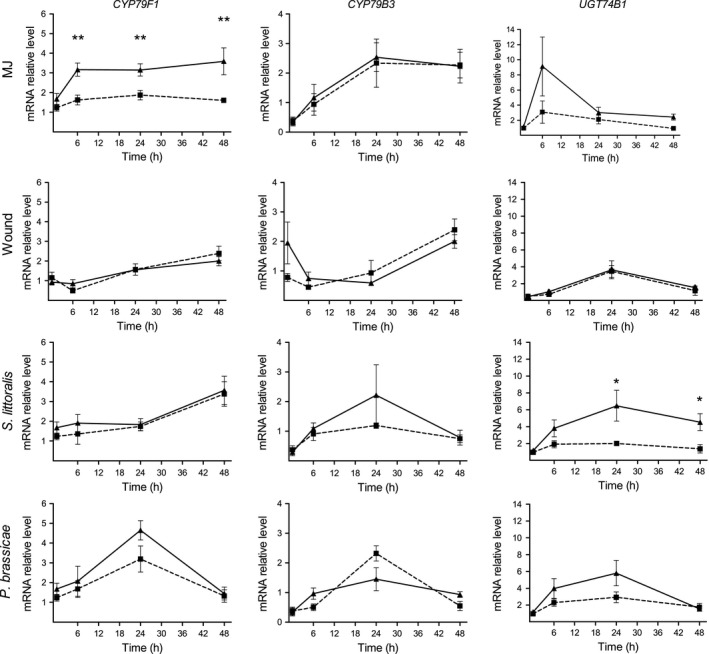
Transcript level of genes involved in the biosynthesis of glucosinolates in leaves of *E. sativa* plants of the two investigated populations: the aliphatic *CYP79F1*, indolic *CYP79B3* and the S‐glycosyltransferase *UGT74B1*. Results present the average ± SE of five biological replicates in plants of the desert (dashed line) and Med (solid line) populations, following induction of defense by MJ, wounding, or herbivory by *S. littoralis* or *P. brassicae* larvae. One and two asterisks represent differential expression between plants of the two populations at *P* < 0.05 and *P* < 0.01, respectively (*t*‐test was applied to log‐transformed data).

Following MJ treatment, significant differences between plants of the two populations were found in the expression of *CYP79F1*, responsible for the first step of aliphatic GS biosynthesis (ANOVA with repeated measures, *F *=* *18.89; *P *<* *0.05; Fig. 5), so that its expression was significantly higher in plants of the Med population than in the desert ones, 6–48 h after treatment (*t*‐tests, *P *<* *0.01). However, no quantitative differences were found between plants of the two population in the expression of *CYP79F1* following wounding or herbivory, either by larvae of the generalist or the specialist insects (Fig. 5). Similarly, no overall differences were found between the two populations in the accumulation of the indolic *CYP79B3* and the postaldoxime *UGT74B1*, in any of the four elicitation treatments (MJ, mechanical wounding or herbivory by generalist and specialist larvae) (ANOVA with repeated measures, *P *>* *0.05; Fig. 5). Nevertheless, significantly higher expression of *UGT74B1* was measured in plants of the Med population than in the desert ones, 24 h (3.2 times) and 48 h (3.3 times) following herbivory by larvae of *S. littoralis* (*t*‐test, *P *<* *0.05).

**Figure 6:**
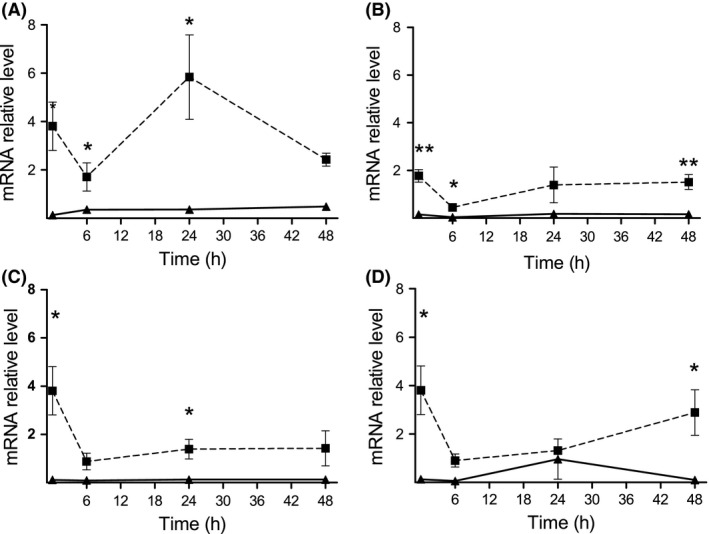
Transcript level of nitrile‐specifier protein (*NSP2*) in leaves of *E. sativa*. Results present the average ± SE of five biological replicates in plants of the desert (dashed line) and Med (solid line) populations following the defense induction by MJ (A), wounding (B) and herbivory by *S. littoralis* (C) and *P. brassicae* (D) larvae. One and two asterisks represent differential expression between plants of the two populations at *P* < 0.05 and *P* < 0.01, respectively (*t*‐test was applied to log‐transformed data).

The expression of the myrosinase‐associated protein *NSP2* was also measured, as this reflects the arrangement of GS breakdown products into simple nitriles (Wittstock and Burow 2007, 2010). ANOVA with repeated measures indicated on overall significant differences between plants of the two populations in its expression following wounding (*F *=* *26.31, *P *<* *0.01), MJ treatment (*F *=* *53.69, *P *<* *0.001) or herbivory by the generalist (*F *=* *26.95, *P *<* *0.01) or specialist (*F *=* *150.91, *P *<* *0.001) herbivores (Fig. 6). Moreover, the noninduced level (control) of *NSP2* was significantly higher in plants of the desert population than in the Med plants (*t*‐test, *P *<* *0.01; Fig. 6).

### Proteinase inhibitor activity

**Figure 7:**
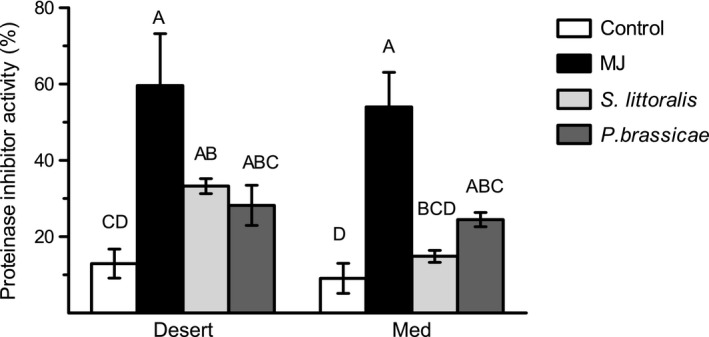
Trypsin PI activity in noninduced and defense‐induced plants of the two populations of *E. sativa*. Enzyme activity was measured 48 h after defense elicitation in leaves by MJ or continuous damage from *S. littoralis* or *P. brassicae* larvae. The results present the average ± SE of five different plants; different letters indicate significant differences between treatments (Tukey's HSD test was applied to log‐transformed data, *P* < 0.05).

Trypsin PI activity (%) was similarly significantly affected in plants of the two populations by either MJ treatment or by the specialist herbivore (Table S3, Fig. 7). In contrast, in response to the generalist herbivore, trypsin PI activity was significantly (2.6 times) induced only in plants of the desert population, in comparison with noninduced plants (Tukey's HSD, *P < *0.05; Fig. 7). Preliminary results indicated that mechanical wounding (Fig. S2) and lanolin (data not shown) did not induce trypsin PI activity in plants of the two populations.

### Expression of genes in the signaling‐regulating pathways

**Figure 8:**
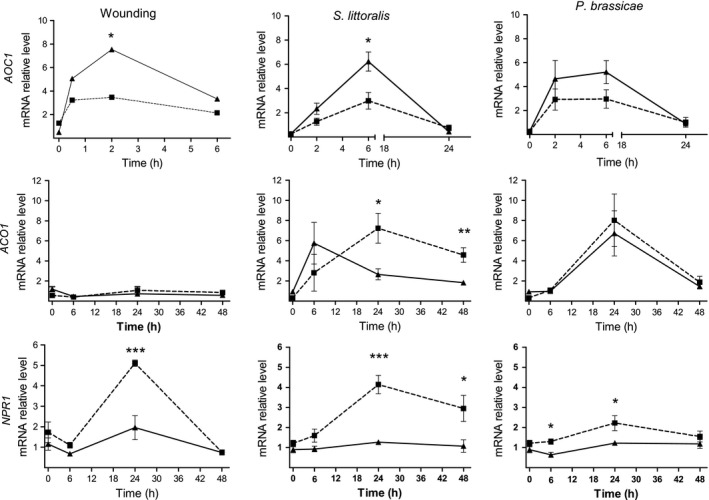
Expression of genes in the signaling‐transduction pathways in plants of the two populations of *E. sativa*: the JA synthesis gene *AOC1*, the ethylene synthesis gene *ACO1*, and *NPR1* as markers for JA–SA interactions. Results present the average ± SE of five biological replicates in plants of the desert (dashed line) and Med (solid line) populations following defense induction by wounding or herbivory by *S. littoralis* or *P. brassicae* larvae. Asterisks present differential expression between plants of the two populations at ^*^ *P* < 0.05, ^**^ *P* < 0.01, and *^**^ *P* < 0.001 (*t*‐test was applied to log‐transformed data).

Following mechanical wounding, significant overall differences were found between plants of the two populations in the accumulation of *AOC1* (ANOVA with repeated measures, *F *=* *95.05, *P *<* *0.01), and 2 h after mechanical wounding, its transcript level was significantly higher (2.9 times) in plants of the Med population than in the desert ones (*t*‐test, *P *=* *0.02; Fig. 8). Herbivory by the generalist and specialist insects had a significant effect on the expression of *AOC1* in plants of the two populations (ANOVA with repeated measures, *F *=* *90.49, *P *<* *0.001 and *F *=* *26.10, *P *=* *0.012, respectively), but 6 h after damage by *S. littoralis*, its expression was significantly higher in plants of the Med population than in the desert plants (*t*‐test, *P *<* *0.05; Fig. 8).

Significant differences between plants of the two populations in the expression of *NPR1* were found after herbivory by the generalist and specialist herbivores (ANOVA with repeated measures, *F *=* *33.71, *P *<* *0.001 and *F *=* *14.74 *P *<* *0.01, respectively), so that higher expression levels were found in plants of the desert population than in the Med ones (*t*‐tests, Fig. 8). Similarly, 24 h following mechanical wounding, the expression of *NPR1* was significantly higher (2.6 times) in plants of the desert population than in the Med plants (*t*‐test, *P *=* *0.01).

The expression of *ACO1* was significantly affected only after herbivory by *P. brassicae* (ANOVA with repeated measures, *F* = 9.8319, *P *=* *0.026). Significant higher transcript levels of *ACO1* were found in plants of the desert population than in the Med ones, 24 h (2.7 times) and 48 h (2.5 times) after damage by the generalist herbivore (*t*‐test, *P *<* *0.05; Fig. 8). But, no overall differences in the expression of *ACO1* were found between plants of the two populations, neither after mechanical wounding nor herbivory (ANOVA with repeated measures, *P *>* *0.05).

## Discussion

Higher expression of *NSP2* was found in plants of the desert population than in plants of the Med population, in both noninduced (control) and when defense was elicited, by either MJ, mechanical wounding, or herbivory (Fig. 6). In the presence of myrosinase, the NSP promotes the formation of simple nitriles from aliphatic and aromatic GS (Burow et al. 2006a) at the expense of ITCs (Wittstock and Burow 2007). It is also known that the bioactive role of GS in the defense against herbivores has been mostly attributed to ITC (Lambrix et al. 2001; Agrawal and Kurashige 2003; Burow et al. 2006b). Accordingly, the lower resistance to *S. littoralis* of noninduced plants of the desert population as compared to the Med ones (Fig. 2A) can be associated to the accumulation of nitriles that are formed upon damage by the larvae.

Induction of plant defense with MJ prior to the feeding assay increased plant resistance to *S. littoralis* larvae, but only in plants of the desert population (Fig. 2A). In addition, no differences were found between plants of the two populations in their defense against *P. brassicae* larvae, or between larvae that were feeding on MJ‐induced and noninduced plants (Fig. 2B). Thus, we analyzed the constitutive and induced levels of GS, assuming that accumulation of these defense compounds in plants of the desert population plays a role in the induced defense response against the generalist insect but not against the GS‐resistant specialist (Fig. 2). In *A. thaliana*, recently published results have demonstrated that larvae of the generalist *Helicoverpa armigera* induce the accumulation of indolic GS, while both indolic and aliphatic GS were induced by larvae of the specialist *Pluttela xylostella* (Badenes‐Perez et al. 2013). Here, larvae of the generalist and specialist insects both induced transcripts accumulation of *CYP79F1* and *CYP79B3* genes, responsible for the first aldoxime formation of aliphatic and indolic GS, respectively (Fig. 5). However, damage by larvae of *S. littoralis* induced the accumulation of *CYP79B3* and the postaldoxime *UGT74B1* only in plants of the Med population. Moreover, MJ treatment significantly increased the accumulation of *CYP79F1* in plants of the Med population, resulting in significant differences between plants of the two populations in its expression level (Fig. 5). Consequently, MJ treatment and damage by both insects was found to significantly increase total GS concentration, but only in plants of the Med population (Fig. 4). GS accumulation can therefore account for the constitutive and induced defense against *S. littoralis* in plants of the Med population.

MJ treatment and damage by *P. brassicae* larvae similarly induced trypsin PI activity in plants of the two populations, but damage by *S. littoralis* larvae significantly induced the protein activity exclusively in plants of the desert population (Fig. 7). PI act in direct defense via suppression of insect growth and development by inhibiting proteases in their guts (Ryan 1990; Zavala et al. 2004). Accordingly, it was anticipated that these antinutritive and starvation effects of PI would not reduce or deter herbivory by the generalist insect as compared to the effect of GS accumulation. Indeed, estimation of plant damage indicated that *S. littoralis* larvae consume similar amounts of plant tissue in non‐induced and MJ‐induced plants of the desert population, while in plants of the Med population, the insect damage was significantly reduced in MJ‐induced plants as compared to the control ones (Fig. 3). Thus, it can be postulated that the induced activity of this antidigestive enzyme in response to damage by *S. littoralis* in plants of the desert population (Fig. 7) is responsible for the increased defense level against the insect (Fig. 2A) and can provide a defense against specialist insects in plants of both populations.

It has been recently shown that the expression of PI in *Arabidopsis* is governed by the complex interactions of phytohormones, in which JA and SA induce PI transcript accumulation, but ethylene suppresses it (Laluk and Mengiste 2011). It has also been shown that herbivory by *S. littoralis* increased PI transcript in *N. attenuata* (Solanaceae) following an induction in SA (Heidel and Baldwin 2004) and in tomato (*Solanum lycopersicum*), the expression of PI genes is regulated both by JA and ethylene (Odonnell et al. 1996). The regulation of GS in *Arabidopsis* is mainly governed by JA and its interaction with ethylene (Mikkelsen et al. 2003; Mewis et al. 2005, 2006), while SA signaling has been shown to suppress GS accumulation (Mewis et al. 2006). In accordance with these reports, we observed differential expression of *NPR1* in response to *S. littoralis* (Fig. 8), a marker gene of antagonistic SA–JA interactions (Spoel et al. 2003), supporting the involvement of SA in negative and positive regulation of GS and trypsin PI, respectively, in plants of the desert population. In addition, wounding induced the accumulation of *AOC1* in plants of the two populations, but not the expression of *ACO1*, suggesting that mechanical damage had no effect on GS concentration (Fig. S1) and trypsin PI activity (Fig. S2) as a result of JA–ethylene interactions. Furthermore, differences in the transcript levels of *AOC1* suggested that in response to wounding and the generalist herbivore, plants of the Med population accumulated elevated levels of JA compared to plants of the desert plants; the expression of *ACO1* suggests that plants of the desert population accumulated higher level of ethylene in response to damage by *S. littoralis* than the Med ones (Fig. 8). Taken together, our results thus suggest that differences between plants of the two populations in their defense against insects can be directly linked to the mode of perceiving herbivory, mediated by the interactions of JA, ethylene, and SA, and consequently resulting in differential regulation of GS and PI in plants of the two populations.

In conclusion, our results indicate intraspecific ecotypic differences in induced defense strategies against generalist herbivores in two populations of *E. sativa*: the accumulation of GS in plants of the Med population and induced activity of trypsin PI in plants of the desert population. For such genetic variation to evolve, natural selection in the two *E. sativa* habitats had to favor local adaptations that have an ecological advantage. Genetic variation in constitutive and induced levels of PI in half‐sib families of *Brassica rapa* has suggested that such natural variation evolved as a result of selection imposed by herbivores (Cipollini et al. 2003). Accordingly, it can be postulated that in plants of the desert population, the induced level of trypsin PI activity provides an effective alternative defense when the hydrolysis of GS is primarily directed to simple nitriles (Fig. 6). It has also been shown that damaged wild‐type *Arabidopsis* plants are more apparent to females of the specialist *Pieris rapae* than lines over‐producing simple nitriles (Mumm et al. 2008). In addition, trichomes on the leaf surface of *B. rapa* have been shown to exert a negative effect on the feeding behavior of larvae of the specialist *P. rapae* (Agren and Schemske 1993). Based on these reports, it is possible that the higher trichome density in plants of the desert population (Westberg et al. 2013) and the arrangement of GS breakdown products to nitriles can have a consequence on plant fitness by reducing damage by specialists. However, more studies are needed to test this hypothesis, by analysis of GS breakdown products, monitoring herbivore communities in the two habitats, and to understand whether these forced evolutionary constraints on defense.

## Data Availability Statement

All relevant data are within the paper and its Supporting Information files.

## Conflict of Interest

The authors have declared that no competing interests exist.

## Supporting information


**Figure S1.** Wounding (A) and lanolin (B) have no effect on total glucosinolate concentration (*μ*mol/g [DW]) in plants of *E. sativa*. Results represent mean ± SE; post‐hoc comparison did not reveal significant differences between the four groups (Tukey HSD, *P *> 0.05).

**Figure S2.** Wounding have no effect on trypsin PI activity* in plants of *E. sativa*. Results represent mean ± SE; post‐hoc comparison did not reveal significant differences between the four groups (Tukey HSD, *P *> 0.05). *Trypsin PI activity was tested here using the agar diffusion assay (van Dam et al. 2001) in reference to the total protein concentration determined according to Bradford (Bradford 1976). Trypsin PI activity was calculated by the clear zone around the tested samples in reference to a standard soybean proteinase inhibitor (Glycine max) curve (Sigma‐Aldrich, Israel), and expressed in nanomole of inhibited trypsin proteinase molecules per milligram of total soluble protein (Jongsma et al. 1994).

**Table S1.** Sequences of primer sets used in the qRT‐PCR analysis.

**Table S2.** Results of two‐way ANOVA assessing the effects of the two populations of *E. sativa* (desert and Med) and the induction treatment (with and without MJ) on growth of larvae of *S. littoralis* and *P. brassicae*, and the damage to plants created by larvae of *S. littoralis*.

**Table S3.** Results of two‐way ANOVA assessing the effects of the induction treatment (MJ, damage by generalist and specialist herbivores) on total GS concentrations and trypsin PI activity in the two populations of *E. sativa* (desert and Med).

**Table S4.** Glucosinolates* (*μ*mol/g DW, mean ± SE) in leaves of *E. sativa*, and 48 h after elicitation with MJ. Different uppercase letters indicate significant differences in each glucosinolate separately (Tukey HSD, *P *< 0.05); values in bold indicate significant differences relative to non‐induced plants. Different superscript letters in a row indicate significant differences at *P *< 0.05. *Glucosativin, 4‐mercaptobutyl GS; glucoraphanin, 4‐methylsulfinylbutyl GS; glucoerucin, 4‐methylthiobutyl GS; glucoraphasatin, 4‐methylthio‐3‐butenyl GS; glucobrassicin, 3‐indolylmethyl GS.

**Table S5.** Glucosinolates* (*μ*mol/g DW, mean ± SE) in leaves of *E. sativa*, and 48 h after continuous elicitation by the specialist (*P. brassicae*) or generalist (*S. littoralis*) herbivores. Different uppercase letters indicate significant differences in each glucosinolate separately (Tukey HSD, *P *< 0.05); values in bold indicate significant differences relative to non‐induced plants. Different superscript letters in a row indicate significant differences at *P *< 0.05. *Glucosativin, 4‐mercaptobutyl GS; glucoraphanin, 4‐methylsulfinylbutyl GS; glucoerucin, 4‐methylthiobutyl GS; glucoraphasatin, 4‐methylthio‐3‐butenyl GS; glucobrassicin, 3‐indolylmethyl GS.
