# Supplementary figures and images for: Intraspecific variation in defense against a generalist lepidopteran herbivore in populations of Eruca sativa (Mill.)
Source: Ecol Evol. 2016 Jan 1;6(1):363–74. doi: 10.1002/ece3.1805 (PMC4716514; doi:10.1002/ece3.1805)

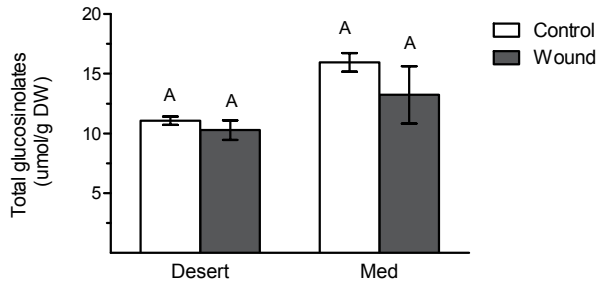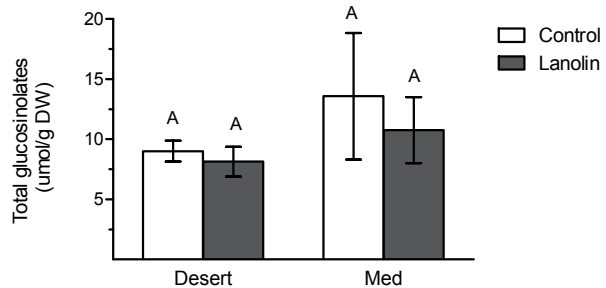

Supplement: Supplementary file 1 — Figure S1. Wounding (A) and lanolin (B) have no effect on total glucosinolate concentration (μmol/g [DW]) in plants of E. sativa. Results represent mean ± SE; post‐hoc comparison did not reveal significant differences between the four groups (Tukey HSD, P > 0.05). [file ECE3-6-363-s001.pdf]

Proteinase inhibitor activity  
(pmol/ug protein )

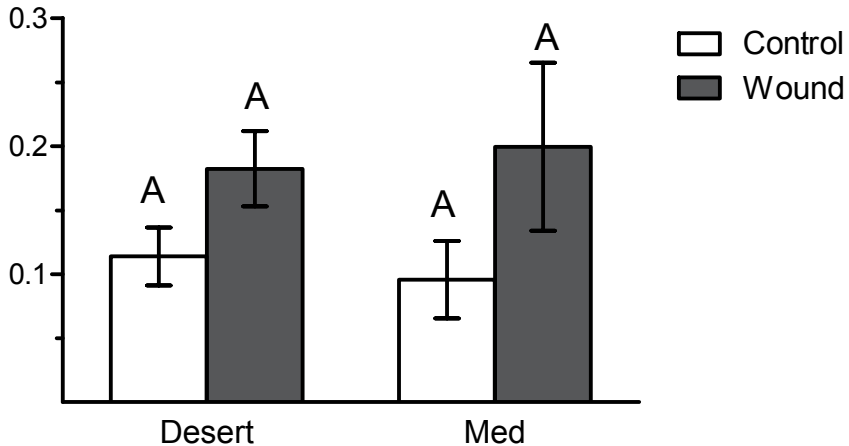

Supplement: Supplementary file 2 — Figure S2. Wounding have no effect on trypsin PI activity* in plants of E. sativa. Results represent mean ± SE; post‐hoc comparison did not reveal significant differences between the four groups (Tukey HSD, P > 0.05). *Trypsin PI activity was tested here using the agar diffusion assay (van Dam et al. 2001) in reference to the total protein concentration determined according to Bradford (Bradford 1976). Trypsin PI activity was calculated by the clear zone around the tested samples in reference to a standard soybean proteinase inhibitor (Glycine max) curve (Sigma‐Aldrich, Israel), and expressed in nanomole of inhibited trypsin proteinase molecules per milligram of total soluble protein (Jongsma et al. 1994). [file ECE3-6-363-s002.pdf]
