# Supplementary material for: Intraspecific variation in defense against a generalist lepidopteran herbivore in populations of Eruca sativa (Mill.)
Source: Ecol Evol. 2016 Jan 1;6(1):363–74. doi: 10.1002/ece3.1805 (PMC4716514; doi:10.1002/ece3.1805)
Supplement: Supplementary file 3 — Table S1. Sequences of primer sets used in the qRT‐PCR analysis. [file ECE3-6-363-s003.docx]

**Table S1**

| **Gene** | **Product size** | **Forward and reverse primers** |
| --- | --- | --- |
| *ACO1* | 161 | ATGACGATGAAGCAGGGAAG  GGGCCTTTGTCTCCAAACTT |
| *AOC1* | 134 | CCCATAATCATCACTCTCATCGAA  TCGAGTAGGGGAGAGATACTTGGA |
| *CYP79B3* | 234 | CAGTGGGTTTCCACTCGTAGGAA  GCCGTTGGAGAGTATCTTTTGAGC |
| *CYP79F1* | 222 | CAATGCGTTGAATTTTGTATAGC  ATGAGCACTTGGGTGAATCC |
| *NPR1* | 104 | CTCGGCTATGAGGTGGCTGA  TGGCCCTCAGTCAAAAGCAT |
| *NSP2* | 118 | TTGACGTGGATCGAAACGTG  ATGGGCTTGAGGGTCCATCT |
| *UGT74B1* | 170 | CTTGGTGTAACCAGCTTGAGG  TTTCCAAACTTCCTCCACAAA |
| Actin | 160 | CTTGGTGCAAGTGCTGTGAT  CCATCGGTGCTGAGAGATTC |
