# Supplementary material for: Intraspecific variation in defense against a generalist lepidopteran herbivore in populations of Eruca sativa (Mill.)
Source: Ecol Evol. 2016 Jan 1;6(1):363–74. doi: 10.1002/ece3.1805 (PMC4716514; doi:10.1002/ece3.1805)
Supplement: Supplementary file 4 — Table S2. Results of two‐way ANOVA assessing the effects of the two populations of E. sativa (desert and Med) and the induction treatment (with and without MJ) on growth of larvae of S. littoralis and P. brassicae, and the damage to plants created by larvae of S. littoralis. [file ECE3-6-363-s004.docx]

**Table S2**

| **Factor** |  | | ***S. littoralis*** | | ***P. brassicae*** | |
| --- | --- | --- | --- | --- | --- | --- |
|  | **df** | ***F* ratio** | | ***P*** | ***F* ratio** | ***P*** |
| Larvae mass |  |  | |  |  |  |
| Population | 1 | 7.8216 | | 0.0129 | 8.9085 | 0.0098 |
| Treatment | 1 | 8.6975 | | 0.0094 | 11.9699 | 0.0038 |
| P×T | 1 | 2.5673 | | 0.1287 | 0.0087 | 0.9271 |
| Herbivore damage | |  | |  |  |  |
| Population | 1 | 32.0203 | | <0.0001 |  |  |
| Treatment | 1 | 38.7385 | | <0.0001 |  |  |
| P×T | 1 | 10.8677 | | 0.0011 |  |  |
