# Supplementary material for: Intraspecific variation in defense against a generalist lepidopteran herbivore in populations of Eruca sativa (Mill.)
Source: Ecol Evol. 2016 Jan 1;6(1):363–74. doi: 10.1002/ece3.1805 (PMC4716514; doi:10.1002/ece3.1805)
Supplement: Supplementary file 5 — Table S3. Results of two‐way ANOVA assessing the effects of the induction treatment (MJ, damage by generalist and specialist herbivores) on total GS concentrations and trypsin PI activity in the two populations of E. sativa (desert and Med). [file ECE3-6-363-s005.docx]

**Table S3**

| **Factor** |  | **MJ** | | ***S. littoralis*** | | ***P. brassicae*** | |
| --- | --- | --- | --- | --- | --- | --- | --- |
|  | **df** | ***F* ratio** | ***P*** | ***F* ratio** | ***P*** | ***F* ratio** | ***P*** |
| Glucosinolates |  |  |  |  |  |  |  |
| Population | 1 | 1.0464 | 0.3192 | 0.1904 | 0.6666 | 0.0016 | 0.9684 |
| Treatment | 1 | 8.3461 | 0.0094 | 12.769 | 0.0016 | 16.5771 | 0.0005 |
| P×T | 1 | 2.7788 | 0.1119 | 0.0903 | 0.7665 | 0.0131 | 0.9100 |
| trypsin-PI |  |  |  |  |  |  |  |
| Population | 1 | 1.0119 | 0.3294 | 6.5213 | 0.0212 | 1.3160 | 0.2682 |
| Treatment | 1 | 36.8384 | <0.0001 | 12.8890 | 0.0024 | 15.5341 | 0.0012 |
| P×T | 1 | 0.7271 | 0.4064 | 0.2266 | 0.6405 | 0.7124 | 0.4111 |
