# Supplementary material for: Intraspecific variation in defense against a generalist lepidopteran herbivore in populations of Eruca sativa (Mill.)
Source: Ecol Evol. 2016 Jan 1;6(1):363–74. doi: 10.1002/ece3.1805 (PMC4716514; doi:10.1002/ece3.1805)
Supplement: Supplementary file 6 — Table S4. Glucosinolates* (μmol/g DW, mean ± SE) in leaves of E. sativa, and 48 h after elicitation with MJ. Different uppercase letters indicate significant differences in each glucosinolate separately (Tukey HSD, P < 0.05); values in bold indicate significant differences relative to non‐induced plants. Different superscript letters in a row indicate significant differences at P < 0.05. *Glucosativin, 4‐mercaptobutyl GS; glucoraphanin, 4‐methylsulfinylbutyl GS; glucoerucin, 4‐methylthiobutyl GS; glucoraphasatin, 4‐methylthio‐3‐butenyl GS; glucobrassicin, 3‐indolylmethyl GS. [file ECE3-6-363-s006.docx]

**Table S4**

| **Glucosinolate^*^** | **Desert** | | **Mediterranean** | |
| --- | --- | --- | --- | --- |
|  | **Control** | **MJ** | **Control** | **MJ** |
| Glucosativin | 3.31±0.51^b^ | 5.07±0.78^ab^ | 3.74±0.31^ab^ | 5.70±0.59^a^ |
| Glucoraphanin | 0.69±0.18^a^ | 0.37±0.04^a^ | 0.49±0.07^a^ | 0.34±0.0^a^ |
| Glucoerucin | 0.23±0.08^a^ | 0.23±0.10^a^ | 0.19±0.07^a^ | 0.27±0.06^a^ |
| Glucoraphasatin | 2.45±0.85^a^ | 3.93±1.10^a^ | 2.73±0.82^a^ | 5.96±1.32^a^ |
| Glucobrassicin | 0.08±0.01^a^ | 0.06±0.02^a^ | 0.06±0.00^a^ | 0.05±0.01^a^ |
| Dimer | 8.35±1.31^a^ | 8.75±1.27^a^ | 7.01±0.51^a^ | 11.27±1.68^a^ |
| X1 | 1.09±0.17^a^ | **0.25±0.06**^b^ | 0.70±0.13^ab^ | 0.47±0.21^b^ |
| Total | 16.20±2.42^ab^ | 18.65±2.64^ab^ | 14.91±0.98^b^ | **24.06±1.67**^a^ |
