# Supplementary material for: Intraspecific variation in defense against a generalist lepidopteran herbivore in populations of Eruca sativa (Mill.)
Source: Ecol Evol. 2016 Jan 1;6(1):363–74. doi: 10.1002/ece3.1805 (PMC4716514; doi:10.1002/ece3.1805)
Supplement: Supplementary file 7 — Table S5. Glucosinolates* (μmol/g DW, mean ± SE) in leaves of E. sativa, and 48 h after continuous elicitation by the specialist (P. brassicae) or generalist (S. littoralis) herbivores. Different uppercase letters indicate significant differences in each glucosinolate separately (Tukey HSD, P < 0.05); values in bold indicate significant differences relative to non‐induced plants. Different superscript letters in a row indicate significant differences at P < 0.05. *Glucosativin, 4‐mercaptobutyl GS; glucoraphanin, 4‐methylsulfinylbutyl GS; glucoerucin, 4‐methylthiobutyl GS; glucoraphasatin, 4‐methylthio‐3‐butenyl GS; glucobrassicin, 3‐indolylmethyl GS. [file ECE3-6-363-s007.docx]

**Table S5**

| **Glucosinolate^*^** | **Desert** | | | **Mediterranean** | | |
| --- | --- | --- | --- | --- | --- | --- |
|  | **Control** | ***P. brassicae*** | ***S. littoralis*** | **Control** | ***P. brassicae*** | ***S. littoralis*** |
| Glucosativin | 3.50 ±0.75^a^ | 5.52±0.65^a^ | 6.06±0.9^a^ | 2.69±0.54^a^ | 5.48±1.14^a^ | 4.71±0.41^a^ |
| Glucoraphanin | 1.47±0.23^a^ | 1.08±0.18^a^ | 0.99±0.22^a^ | 1.03±0.23^a^ | 0.98±0.15^a^ | 1.40± 0.23^a^ |
| Glucoerucin | 0.04±0.02^a^ | 0.24±0.13^a^ | 0.12±0.04^a^ | 0.18±0.03^a^ | 0.09±0.04^a^ | 0.23± 0.09^a^ |
| Glucoraphasatin | 2.79±0.66^bc^ | 10.12±1.71^ab^ | 5.52±2.42^abc^ | 1.67±0.60^c^ | 8.63±1.15^abc^ | **11.74±2.73**^a^ |
| Glucobrassicin | 0.08±0.01^a^ | 0.08±0.01^a^ | 0.09±0.01^a^ | 0.08±0.01^a^ | 0.10±0.01^a^ | 0.08±0.02^a^ |
| Dimer | 9.62±1.07^a^ | 12.95±2.29^a^ | 15.48±1.90^a^ | 9.39±1.16^a^ | 14.40±1.91^a^ | 12.85±1.57^a^ |
| X1 | 1.05±0.13^a^ | 0.71±0.05^a^ | 1.10±0.12^a^ | 1.17±0.17^a^ | 0.80±0.11^a^ | 0.77±0.22^a^ |
| Total | 18.54±2.00^bc^ | 30.70±3.82^ab^ | 29.35±4.49^abc^ | 16.21±3.21^c^ | **30.48±2.52**^ab^ | **31.78±3.38**^a^ |
